# Management of Sickle Cell Disease Super Utilizers

**DOI:** 10.5811/westjem.2016.12.33224

**Published:** 2017-01-30

**Authors:** Gary A. Johnson

**Affiliations:** * SUNY Upstate Medical University, Department of Emergency Medicine, Syracuse, New York

Much attention has been directed toward super utilizers of emergency department (ED) and hospital services. Often these patients have a chronic illness with significant potential for acute morbidity. In many settings, adults with sickle cell disease (SCD) are a significant proportion of super utilizers. This population has a significantly shortened life span compared to other adults as well as a high morbidity including acute life-threatening diseases such as acute chest syndrome and stroke. Compared to other chronic diseases, SCD patients have significantly higher^1^ admission and readmission rates, and outpatient resources are often poorly available.^2^

Quality and uniformity of care across clinical locations is often questioned. Recent authors have highlighted that there are significant differences between specialists in the approach to pain management with vaso-occlusive crisis. This includes differences between hospitalists trained in internal medicine and hematologists.^3^ Other authors have highlighted the difficulty of consistently providing high quality education to sickle-cell patients and their families. High utilizers of hospital services are often characterized by significant social and psychiatric challenges both in the SCD patient and in the supporting family.^4^

In this edition of *Western Journal of Emergency Medicine*^5^ Simpson et. al. describe an intervention to enroll ED super utilizers with SCD in an ED management protocol and the formation of a medical home. The effort required for this intervention is significant and needs to be emphasized. This multidisciplinary clinic included a primary care doctor, social worker, addiction and pain specialist, pharmacist and psychologist. They demonstrated that ED utilization and length of stay, as well as admission rate and inpatient length of stay, can all be decreased using this method. Mortality and ICU readmission did not occur in the study group, but the small sample prevents an adequate statistical analysis. Such a targeted approach, which coordinates ED, inpatient and outpatient settings, is ideal for managing a chronic illness with significant potential for acute morbidity.

Other authors have highlighted the need for coordinated care and alternatives for ED management of exacerbations of SCD. Alternatives should be prompt and available a large number of hours to sufficiently replace the convenient 24/7 access of the ED.^6^ The level of care must be appropriate for any reasonable acute exacerbation of SCD. Specialty infusion centers have been proposed by a large number of authors^7^ and have demonstrated significant decrease in admission rates. Such centers require individual care plans, and support from social services and providers who are comfortable with SCD. Telemonitoring^8^ has been advocated as a method of helping providers get access to expert opinion for their individual SCD patients. Continuing medical education on SCD and appropriate support for such providers may allow a larger number of providers to step into this critical gap of support of outpatient care.

EDs provide a life-saving environment for chronically ill patients with acute exacerbation of illness. EDs also provide an opportunity to treat the patient in accordance with a consistent care plan that is shown to decrease morbidity as well as resource utilization. The article by Simpson et. al.^5^ describes a process that requires a significant investment of clinical resources but also a significant improvement in resource utilization. With larger numbers of participants, it may be possible to achieve cost savings through economies of scale. This approach can be replicated for patients with SCD as well as other resource-intensive chronic illnesses (for example, heart failure or advanced chronic obstructive pulmonary disease). As payers change from fee for service to population health models of reimbursement, EDs will have opportunities to participate in more multidisciplinary chronic care plans.
